# Self‐assembled human placental model from trophoblast stem cells in a dynamic organ‐on‐a‐chip system

**DOI:** 10.1111/cpr.13469

**Published:** 2023-05-17

**Authors:** Rongkai Cao, Yaqing Wang, Jiayue Liu, Lujuan Rong, Jianhua Qin

**Affiliations:** ^1^ Division of Biotechnology Dalian Institute of Chemical Physics, Chinese Academy of Sciences Dalian China; ^2^ University of Chinese Academy of Sciences Beijing China; ^3^ School of Biomedical Engineering University of Science and Technology of China Hefei China; ^4^ Suzhou Institute for Advanced Research University of Science and Technology of China Suzhou China; ^5^ Kunming University of Science and Technology Kunming China; ^6^ Beijing Institute for Stem Cell and Regeneration, Chinese Academy of Sciences Beijing China

## Abstract

The placental barrier plays a key role in protecting the developing fetus from xenobiotics and exchanging substances between the fetus and mother. However, the trophoblast cell lines and animal models are often inadequate to recapitulate the key architecture and functional characteristics of human placental barrier. Here, we described a biomimetic placental barrier model from human trophoblast stem cells (hTSCs) in a perfused organ chip system. The placental barrier was constructed by co‐culture of hTSCs and endothelial cells on the opposite sides of a collagen‐coated membrane on chip. hTSCs can differentiate into cytotrophoblasts (CT) and syncytiotrophoblast (ST), which self‐assembled into bilayered trophoblastic epithelium with placental microvilli‐like structure under dynamic cultures. The formed placental barrier displayed dense microvilli, higher level secretion of human chorionic gonadotropin (hCG), enhanced glucose transport activity. Moreover, RNA‐seq analysis revealed upregulated ST expression and activation of trophoblast differentiation‐related signalling pathways. These results indicated the key role of fluid flow in promoting trophoblast syncytialization and placental early development. After exposure to mono‐2‐ethylhexyl phthalate, one of the endocrine disrupting chemicals, the model showed inhibited hCG production and disturbed ST formation in trophoblastic epithelium, suggesting impaired placental structure and function elicited by environmental toxicants. Collectively, the hTSCs‐derived placental model can recapitulate placenta physiology and pathological response to external stimuli in a biomimetic manner, which is useful for the study of placental biology and associated diseases.

## Graphical abstract

Human placental barrier is consisted of trophoblastic layer, basal lamina, and the fetal capillaries in vivo. We developed a biomimetic placental barrier‐on‐a‐chip model by self‐assembly of human trophoblast stem cells in a dynamic microenvironment, recapitulating the key architecture and functions of human placental villi.

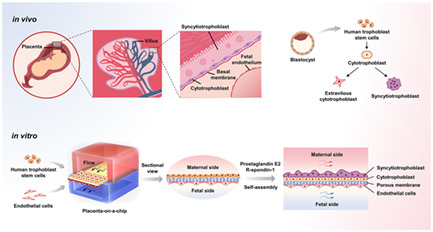

## 1 INTRODUCTION

**FIGURE 1:**
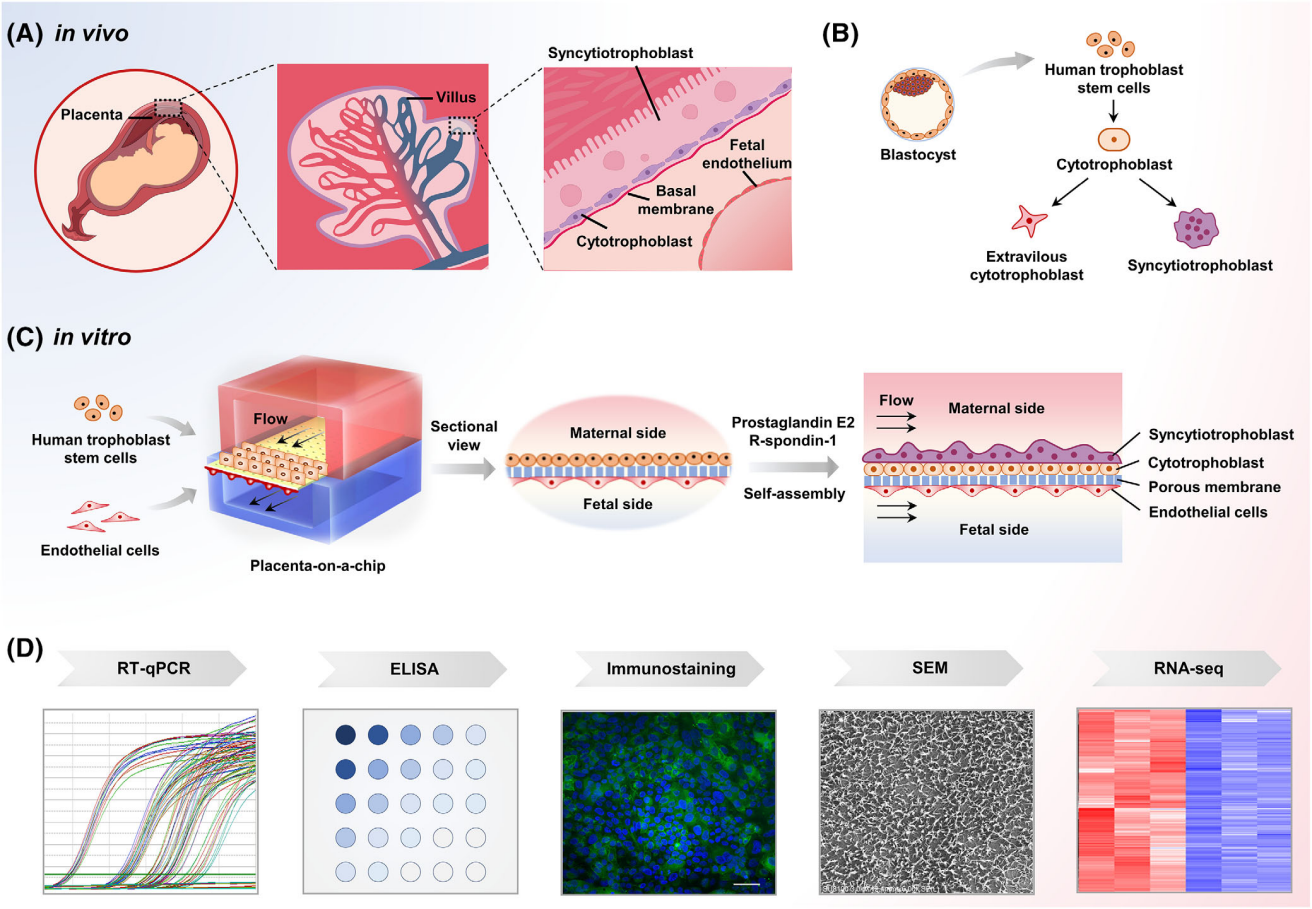
Schematic of the biomimetic placental barrier model derived from hTSCs. (A) The placenta is located at the interface between maternal and fetal blood. Human placental barrier is composed of fetal endothelial layer, basal membrane and trophoblastic layers in vivo. Trophoblastic epithelium in early pregnancy consists of the ST layer and the underlying CT layer. (B) The hTSCs were derived from trophectoderm of blastocysts, which can differentiate into three major trophoblast lineages (CT, ST and EVT). (C) A bioengineered placental barrier model was constructed in a perfused organ‐on‐a‐chip system. hTSCs were seeded on the upper channel to form the bilayered trophoblastic epithelium. HUVECs were cultured on the other side of collagen‐coated membrane to mimic the fetal endothelium. (D) The structure and function of placenta‐on‐a‐chip model were characterized with different methods.

The placental barrier is an essential interface to maintain normal pregnancy and support fetal development between the mother and fetus.^1^, ^2^ In the first trimester, the placental barrier is composed of multilayered structure including the mononuclear cytotrophoblast (CT), the syncytiotrophoblast (ST), basal lamina, and the fetal capillaries (Figure 1A). It can not only supply essential nutrient substance, such as glucose, amino acids and vitamins for fetal growth, but also plays a key role in protecting fetus from exposure of virus, bacteria and xenobiotics.^2^, ^3^, ^4^ The pathogens or noxious chemicals in maternal blood may pass through the placental barrier and disturb fetal development during early pregnancy. The study of impact of exogenous harmful agents on human placental barrier is instructive for reproductive health.

Currently, various experimental models have been developed to study the response of human placenta to external stimulus. Generally, animal models have great variations in the architecture of placenta from that in humans due to species differences.^5^, ^6^, ^7^ The isolated explant cultures or primary placental cells could mimic the complex composition and structure of human placenta,^8^, ^9^, ^10^ but the use of human tissue samples is limited due to ethical concerns of tissue availability, storage and manipulation. In vitro trophoblast cell culture models, such as BeWo^11^, ^12^ and Jeg‐3^13^, ^14^ cell lines and human induced pluripotent stem cells (hiPSCs)^15^ have been used to construct placental barrier, but they are often inadequate to recapitulate the complex placental architectures and functions due to abnormal cell phenotype or immature functions of trophoblasts. Recently, human trophoblast stem cells (hTSCs) were obtained from primary placental tissues or blastocysts.^16^ hTSCs have the capacity to give rise to three major trophoblast subpopulations, including CT, ST and extravillous cytotrophoblast (EVT) (Figure 1B). It provides a valuable cell source for placenta research. Trophoblast organoids were recently established from hTSCs to recapitulate placental development and function.^17^, ^18^ However, organoid models are often limited to recreate the multilayered structure and functions of placental barrier due to the lack of endothelium or dynamic microenvironment.

Organ‐on‐a‐chip (organ chip) is a microfluidic cell culture device that allows to recapitulate the key architectural and functional hallmarks of living organs in vitro.^19^, ^20^ It can mimic the in vivo‐like cellular microenvironment by control over tissue‐tissue interface, dynamic flow and biochemical signals. Advances in organs‐on‐chips have enabled the creation of various models of human organs, such as the lung, gut, liver and so on.^20^, ^21^, ^22^, ^23^ Although several placental barrier‐on‐a‐chip models have been reported previously,^11^, ^12^, ^14^, ^24^ these models were commonly established using trophoblast carcinoma cell lines, which do not represent the near physiological features of human placenta.

In this work, we described a new strategy to create a 3D biomimetic placental model from hTSCs by combining stem cell biology and organ chip technologies. The hTSCs were derived from trophectoderm cells of the human blastocyte based on the published protocol.^16^ The chip device consisted of upper and lower culture chambers incorporated with fluid flow that allowed the formation of placental barrier by coculture of hTSCs and human umbilical vein endothelial cells (HUVECs). hTSCs can differentiate into CT and ST with differentiation medium, which assembled into bilayered trophoblastic epithelium under fluid flow. The formed placental barrier was characterized by cell morphology, expressions of trophoblast‐specific genes and proteins as well as the production of hormone using real‐time qPCR, ELISA and immunofluorescence analysis (Figure 1D). The effects of fluid flow on the differentiation of trophoblastic epithelium from hTSCs were assessed by transcriptome analysis. In addition, the responses of this placental barrier on chip to phthalates were evaluated, indicating the utility of this new model system in the study of placental biology and associated diseases.

## 2 MATERIALS AND METHODS

### 2.1 Human sample

Human blastocyst was obtained with signed informed consent of the donors, and the approval of the Medicine Ethics Committee of The First People's Hospital of Yunnan Province (2017LS[K]NO.035).^25^ Based on the guiding principles in the International Society for Stem Cell Research (ISSCR), written informed consent was obtained from all donor couples for voluntary donations of embryos.

### 2.2 Cell culture

The hTSCs were established from human blastocysts according to the published protocol.^16^ hTSCs were cultured on Collagen IV‐coated plates and maintained in TS medium containing DMEM/F12 basic medium (Gibco), 0.1 mM 2‐mercaptoethanol (Sigma), 0.2% FBS, 0.5% Penicillin–Streptomycin, 0.3% HSA, 1% ITS‐X supplement (Sigma), 1.5 mg/mL L‐ascorbic acid, 50 ng/mL EGF (PeproTech), 2 mM CHIR99021 (Selleckchem), 0.5 mM A83‐01 (Sigma), 1 mM SB431542, 0.8 mM VPA and 5 mM Y27632 (Selleckchem). HUVECs were isolated from human umbilical vein. HUVECs were cultured on Collagen I‐coated plates and maintained in endothelial cell medium (ECM) (Sciencell).

### 2.3 Microchip fabrication

The organ chip was fabricated through standard soft lithography techniques as described in our previous study.^26^ Briefly, moulds made of SU‐8 (MicroChem Corp.) were prepared using photolithography. Then, polydimethylsiloxane (PDMS) (Dow Corning) mixed with monomer and curing agent at a ratio of 10:1 was poured on the moulds. After incubation at 80°C for 30 min, the upper and lower layers with microchannels of 20 mm (length) × 1.2 mm (width) × 0.2 mm (height) were prepared. For assembly of the chip, PET nuclear pore membrane with 2 μm pores was sandwiched between the upper and lower layers following the treatment of plasma.

### 2.4 Construction of placental barrier on chip

The assembled chip was sterilized by UV irradiation overnight. Then, microchannels were coated with Collagen IV (Corning, 0.01 mg/mL in DPBS) at 37°C for 6 h. hTSCs were first seeded on the upper microchannel at a concentration of 6 × 10^6^ cells/mL. TS medium was changed every 12 h to maintain hTSCs proliferation for 2 days. Next, HUVECs at a concentration of 2 × 10^6^ cells/mL were introduced into the lower microchannel. The chip was then inverted and incubated at 37°C for 2 h. Following the attachment of HUVECs, ECM medium was perfused into the lower microchannel by syringe pump with a speed of 10 μL/h. Meanwhile, hTSCs differentiation medium containing DMEM/F12 basic medium, 0.5% N2 supplement, 1% B27 supplement minus vitamin A, 100 μg/mL Primocin, 1.25 mM N‐Acetyl‐L‐cysteine, 2 mM L‐glutamine, 50 ng/mL EGF, 1.5 μM CHIR99021, 80 ng/mL R‐spondin‐1 (R&D systems), 100 ng/mL FGF‐2 (PeproTech), 50 ng/mL HGF (PeproTech), 500 nM A83‐01, 2.5 μM prostaglandin E2 (Sigma) and 2 μM Y‐27632 was injected into upper microchannel at 10 μL/h (low FSS) or 100 μL/h (high FSS). For static culture group, ECM and differentiation medium were changed every 12 h. After cocultivation of hTSCs and HUVECs for another 4 days, the placenta barrier model was constructed.

### 2.5 Mono‐2‐ethylhexyl phthalate (MEHP) treatment

Following the construction of placental barrier model, trophoblast cells in the upper microchannel were treated with differentiation medium containing 1 μM MEHP (BGB Analytik AG) or vehicle (DMSO, Sigma) for 48 h under static culture. Cell medium was changed every 12 h.

### 2.6 Permeability assay

FITC‐labelled dextran (*M*_w_ = 70 kDa) was used in permeability assay to exam the integrity of the placental barrier model. 3 μM FITC‐dextran in DMEM/F12 basic medium was perfused into the upper microchannel. Then, the perfusate from the lower microchannel was collected every 30 min for 3 h and added into a black 96‐well plate after dilution. Fluorescence intensity was assessed by a microplate reader (Tecan) and the concentration was determined using a dilution standard curve.

### 2.7 Scanning electron microscopy

Samples were fixed with 2% glutaraldehyde and 1% osmic acid successively for 2 h at room temperature. Then, the fixed samples were dehydrated with a series of diluted ethanol, substituted with isoamyl acetate, dried using critical point dryer (Quorum) and sputter‐coated with gold in ion sputtering apparatus (Hitachi). Next, samples were observed and imaged using a scanning electron microscopy (SU8100, Hitachi).

### 2.8 Immunofluorescence

Cells on the chip were fixed in 4% paraformaldehyde for 20 min at room temperature. Next, microchannels were washed with DPBS and filled with 0.2% Triton X‐100 for 10 min. Then, samples were blocked with goat serum for 1 h and incubated with primary antibody at 4°C overnight. Primary antibodies used in the experiment were listed as follow: CDH1 (mouse, CST, 1:100), CGB (rabbit, Abcam, 1:300), CK7 (mouse, Invitrogen, 1:100), GATA3 (mouse, Santa Cruz, 1:100), GLUT1 (rabbit, CST, 1:100), VE‐cadherin (rabbit, CST, 1:100), PLIN2 (rabbit, Abcam, 1:500). After washed with DPBS, samples were incubated with Alexa Fluor 488‐ or 594‐conjugated secondary antibodies (CST, 1: 500) at room temperature for 1 h and then stained with DAPI (CST, 1:4000) for 10 min. For F‐actin staining, samples were stained with Alexa Fluor 488‐conjugated phalloidin (Biotium) for 20 min at room temperature according to the manufacture' s instruction. The stained samples were imaged using a confocal microscope (FV3000, Olympus).

### 2.9 Real‐time quantitative PCR

Total RNA was isolated from cells on the chip using Trizol reagent (TAKARA). RNA quality and concentration were determined by NanoPhotometer (IMPLEN). cDNA was produced after RNA was diluted to 50 ng/μL. Then, cDNA was amplified via qPCR using Ex Taq DNA polymerase (TAKARA) under the following reaction conditions (40 cycles): denaturation at 94°C for 1 min, annealing at 58°C for 45 s, and extension at 72°C for 30 s. The primer pairs used are listed in Table S1 (Supplementary Material). GAPDH was used as a reference gene in each sample.

### 2.10 ELISA

Human chorionic gonadotropin (hCG) ELISA kit (Cloud‐Clone) was used to examine the hCG secreted from trophoblast cells. After construction of the placental barrier model or treatment of MEHP, cell medium was changed and maintained under static culture for another 12 h. Then, the medium was collected and stored at −80°C until use. For detection of hCG concentration, samples were prepared following the instructions provided by manufacturer and the fluorescence intensity was assessed by a microplate reader.

### 2.11 Oil red O staining

Trophoblast cells on the chip under static or dynamic culture were fixed in 4% paraformaldehyde for 20 min at room temperature. Next, microchannels were washed with DPBS and filled with 0.2% Triton X‐100 for 10 min. Then, the lipid droplets in trophoblasts were stained by Oil red O (Sigma‐Aldrich) for 20 min. After the remaining dye was washed away, the stained samples were imaged using a fluorescence microscope (Olympus).

### 2.12 2‐NBDG based assays

The hTSCs were cultured on the chip with or without differentiation medium perfusion for 4 days. After the microchannels were washed by DPBS, the maternal side was perfused with 1 mM 2‐NBDG (Psaitong). Next, the chip devices were incubated at 37°C for 30 min. Then, the buffer in fetal channel was collected and trophoblast cells were lysed with 1% Triton X‐100 for 5 min. Fluorescence intensity of the buffer and lysis solution were analysed by a microplate reader (Tecan).

### 2.13 RNA sequencing

Trophoblast cells on the chip under static or dynamic culture were collected after treated with differentiation medium for 3 days. Total RNA was isolated from samples using Trizol reagent. Then, 2 μg RNA was used for standard RNA sequencing library preparation with Ribo‐off rRNA Depletion Kit (Illumina) and KC‐Digital™ Standard mRNA Library Prep Kit for Illumina (Seqhealth) following the instructions provided by manufacturer. The library products corresponding to 200–500 bps were enriched, quantified and sequenced on NovaSeq 6000 sequencer (Illumina) with PE150 model. Sequencing data were analysed through standard RNA‐seq protocol. Reads were mapped to the reference genome of *Homo sapiens* from Homoserines (GRCh38) using STAR software (version 2.5.3a) with default parameters.

### 2.14 Cell viability analysis

hTSCs and HUVECs were cultured in a 96‐well plate and exposed to 0 (vehicle), 0.1, 1, 10, 50, 100 μM MEHP for 48 h. Then, the viability of cells was measured with calcein‐AM (Beyotime) following the manufacturer's instructions. The fluorescence intensity was assessed by a microplate reader and the percentage of cell viability was determined by comparation with blank control.

### 2.15 Statistical analysis

Data were expressed as the mean ± standard error of the mean (SEM) or mean ± standard deviation (SD) for at least three independent experiments. The difference between two groups was analysed using Student's *t*‐test. Significance was indicated by asterisks: *p < 0.05; **p < 0.002; ***p < 0.001. The image‐based fluorescence intensity statistics were sampled from three different chips and each for three different areas. The areas were picked randomly.

## 3 RESULTS

### 3.1 Self‐assembled bilayered trophoblastic epithelium from hTSCs in a perfused chip system

In vivo, human placenta barrier is consisted of the maternal facing trophoblastic epithelium layer, connective tissue and endothelium lining the fetal capillaries in the first trimester. After implantation, villous CT cells proliferate and fuse to form the multinucleated ST, which assembles into the trophoblastic epithelial layer of the chorionic villi exposed to blood flow.^27^ hTSCs derived from the blastocyst can differentiate into major trophoblast subtypes including CT and ST.^16^ In order to build the biomimetic human placental barrier in vitro, we initially identified the formation of trophoblastic epithelium from hTSCs in a dynamic microenvironment.

**FIGURE 2:**
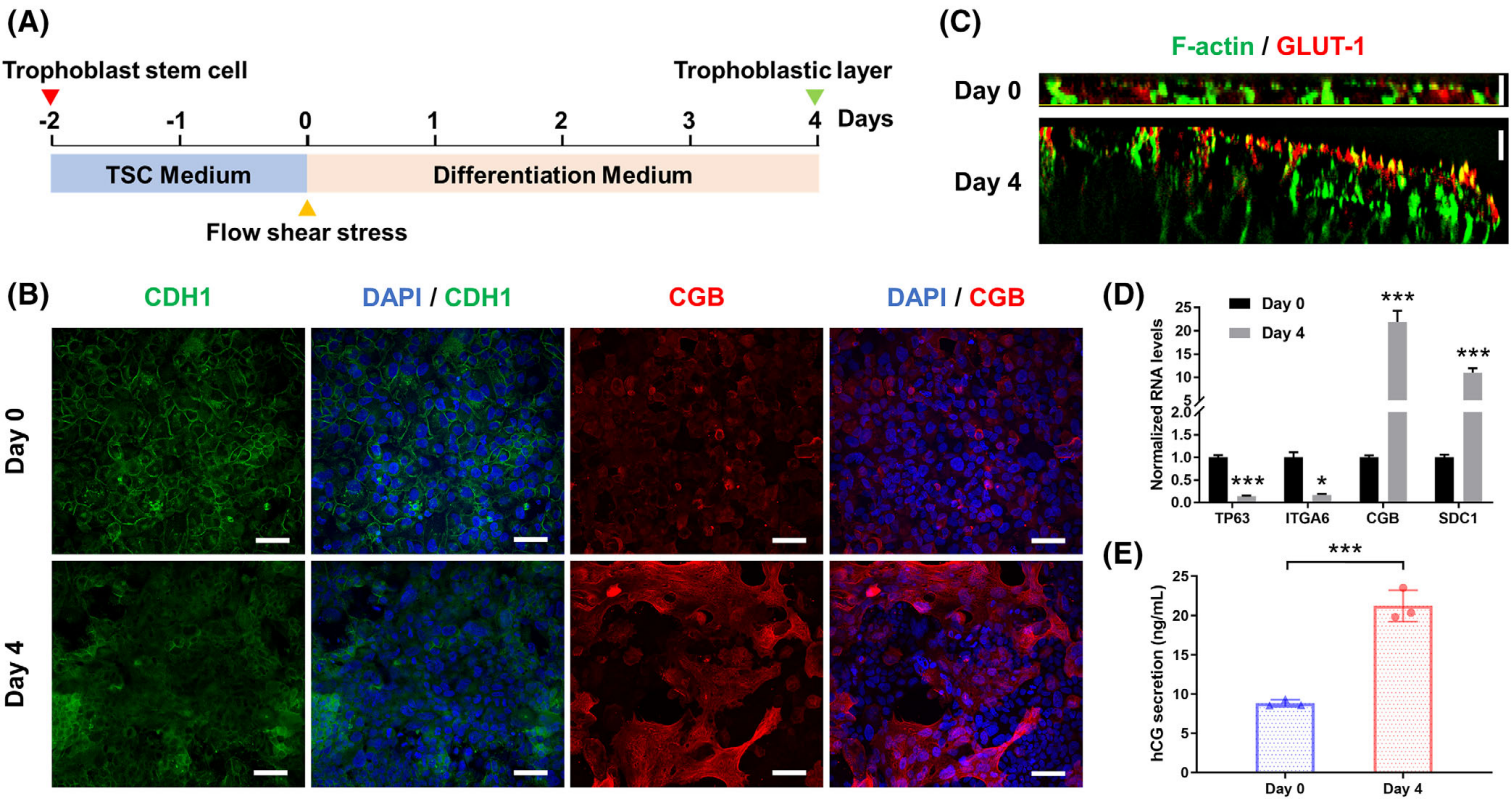
Differentiation of hTSCs in the perfused chip. (A) Experimental workflow of the trophoblastic barrier construction. (B) Representative fluorescence image of the trophoblast layer stained with CDH1 antibody (green), CGB antibody (red) and DAPI (blue) on day 0 or day 4. Scale bars are 50 μm. (C) Representative fluorescence image of x–z optical sections of trophoblast cells stained with GLUT1 antibody (red) and phalloidin (F‐Actin, green). Scale bars are 5 μm. (D) Relative mRNA expression of TP63, ITGA6, CGB and SDC1 in trophoblast cells collected on day 0 and day 4. mRNA expression normalized to GAPDH RNA level was analysed by real‐time PCR. The data are presented as the mean ± SEM from three independent experiments. Data significance was assessed by unpaired two‐tailed Student's *t*‐test; *p < 0.05, ***p < 0.001. (E) hCG secretion by trophoblast cells on day 0 and day 4. The concentration of hCG in culture medium was analysed by ELISA kit. The data are presented as the mean ± SD from three independent experiments. Data significance was assessed by unpaired two‐tailed Student's *t*‐test; ***p < 0.001.

Herein, we designed a perfusable chip device comprised of multilayered channels separated by a porous membrane. The multilayered design simulated the microscopic structure of human placenta at the fetal‐maternal interface. hTSCs were seeded on the upper side of the Collagen IV‐coated membrane of the chip to mimic the maternal side under continuous media flow (10 μL/h). The flow resembled the dynamic blood environment in vivo. The differentiation process of hTSCs on chip is shown in Figure 2A. During the period of hTSCs differentiation, the adherens junction (CDH1) of trophoblasts was analysed by immunofluorescence at different time points. The result showed clear expression of CDH1 in the undifferentiated hTSCs layer on day 0, while the decreased and discontinuous expression on the trophoblast layer after 4 days of differentiation. It indicated the loss of epithelial intercellular junctions and the fusion of trophoblast cells. Moreover, the increased expression of a typical ST marker (CGB) was examined in the trophoblast layer on day 4, demonstrating the syncytialization of hTSCs (Figures 2B and S1). In addition, the trophoblast epithelium showed downregulation of CT markers (TP63 and ITGA6) and upregulation of ST markers (CGB and SDC1) on day 4 examined by RT‐qPCR, indicating the reduction of stemness and syncytialization of trophoblast cells during the differentiation process (Figure 2D).

Glucose transfer across placental barrier via GLUTs is required for fetal development. GLUT1 is the most abundant type of glucose transporters that presents on the surface of placental microvilli.^28^ The fluorescence image of x‐z optical sections of placental barrier demonstrated the increased expression of GLUT1 in the apical membrane of trophoblast epithelium after 4 days of differentiation(Figure 2C). Furthermore, GLUT1 was mostly located on the apical membrane of trophoblasts on day 4 while randomly on day 0. Besides, F‐actin cytoskeleton stain showed the increased thickness of trophoblast cell layer after differentiation (Figures 2C and S2). Our model recapitulated the bilayered structure of trophoblastic epithelium and spatial distribution of GLUT1 as like in vivo status of first‐trimester human placenta.

The placental hormone production is an important functional consequence of trophoblast syncytialization.^29^ The secretion of human chorionic gonadotrophin (hCG), one of the typical placental hormones, was examined in trophoblast cells on days 0 and 4 by ELISA. The result showed significantly increased production of hCG in trophoblasts on day 4 compared with the undifferentiated hTSCs, which further proved the efficient differentiation of hTSCs in the perfused chip system(Figure 2E).

### 3.2 Construction of functional 3D human placental barrier on chip

**FIGURE 3:**
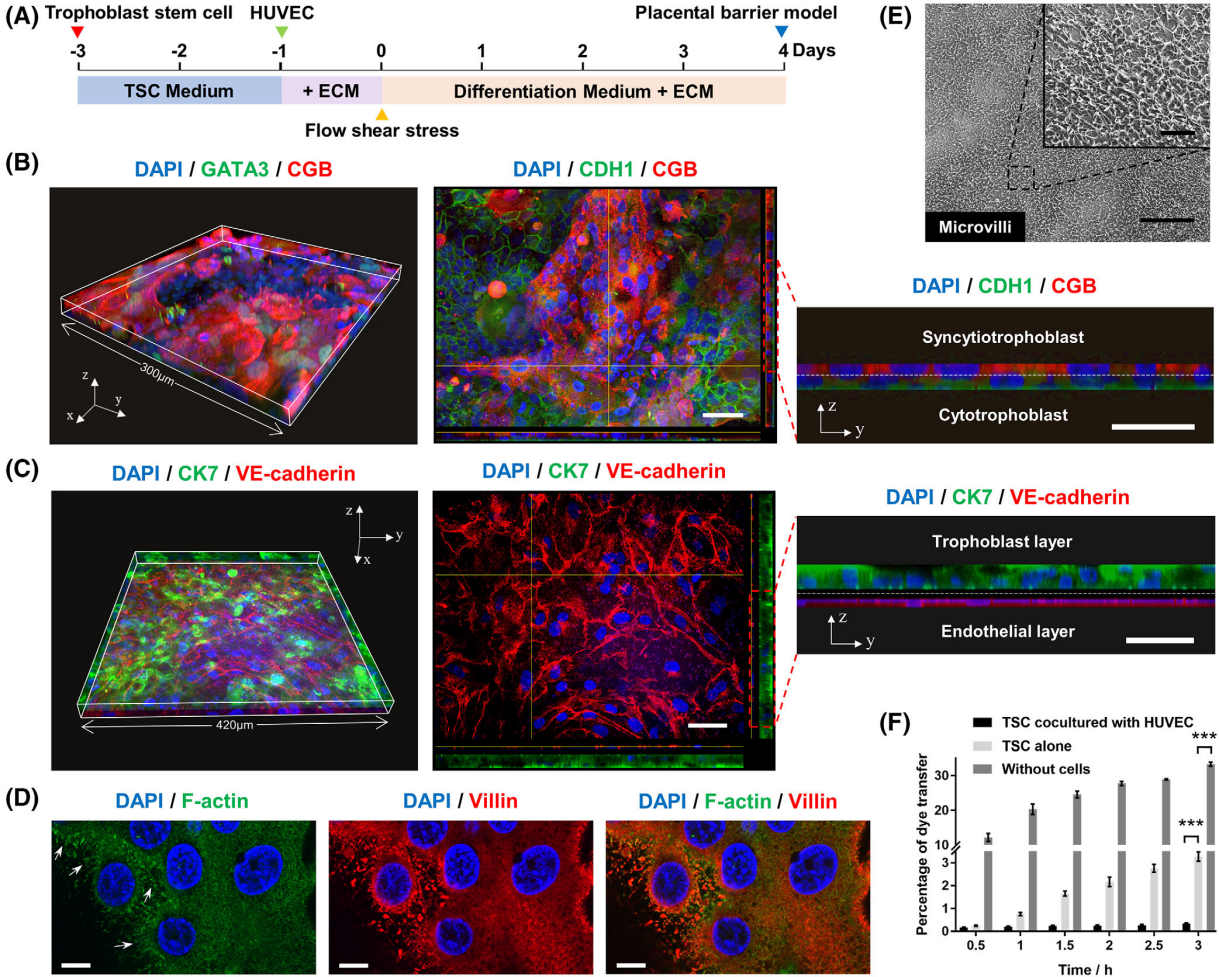
Characterization of hTSC‐derived placental barrier‐on‐a‐chip model. (A) Experimental workflow of the placental barrier model construction. (B) 3D reconstruction of the trophoblastic epithelium stained with CGB antibody (red), GATA3 antibody (green) and DAPI (blue) (left). And representative three sides image of the trophoblastic epithelium stained with CGB antibody (red), CDH1 antibody (green) and DAPI (blue) (right). Scale bars are 50 μm and 30 μm (magnified view of y‐z plane). (C) 3D reconstruction of the placental barrier model stained with VE‐cadherin antibody (red), CK7 antibody (green) and DAPI (blue). And representative three sides image of the placental barrier stained with VE‐cadherin antibody (red), CK7 antibody (green) and DAPI (blue) (right). Scale bars are 50 μm and 30 μm (magnified view of y‐z plane). (D) Representative fluorescence image of the trophoblastic barrier stained with Villin antibody (red), phalloidin (F‐Actin, green) and DAPI (blue). Scale bars are 10 μm. (E) Representative scanning electron microscopic image on the surface of the trophoblastic barrier. Scale bars are 15 μm and 3 μm (enlarged area). (F) Percentage of dye transfer from the upper channel to the lower channel in 0.5, 1, 1.5, 2, 2.5 and 3 h. The data are presented as the mean ± SEM. Data significance was assessed by unpaired two‐tailed Student's *t*‐test; ***p < 0.001.

wTo enable the construction of multilayered placental barrier, hTSCs and HUVECs were cocultured on the opposite sides of collagen‐coated membrane under continuous media flow, thus forming the placental epithelium‐endothelium tissue interface (Figure 3A). As shown in the immunostaining images through z‐axis scanning and 3D reconstruction, the hTSC‐derived trophoblastic epithelium exhibited the marked expression of CT marker (CDH1), ST marker (CGB) and nonspecific trophoblast marker (GATA3). The x‐y plane of three views displayed the visible result of z‐projection by max intensity. The x‐z and y‐z section showed the bilayered structure composed of ST layer and CT layer, which was similar to the spatial structure of placental trophoblasts in vivo^30^ (Figure 3B). The structural integrity of the placental barrier was assessed by the formation of intercellular junctions. Immunostaining images of the multilayered placental barrier showed the clear expression of CK7 in the trophoblast layer and VE‐cadherin in endothelial layer (Figure 3C and Supplementary Figure 1B). The x‐y plane of three views displayed the planer structure of vascular network in the lower channel. The x‐z and y‐z section showed the HUVECs and trophoblast layers separated by the basal membrane.

We further examined the formation of microvilli on the trophoblast layers. Microvilli are typical structural feature of placental syncytium, which can increase the surface area of cells and facilitate functions in absorption, secretion and cellular adhesion.^31^ According to the previous report, fluid flow is an important external cue for microvilli formation.^32^ In this model, the microvilli‐like structure was observed on the apical membrane of the trophoblast layer identified by F‐actin and Villin staining (Figure 3D). The co‐localization of F‐actin and Villin indicated the maturation of ST. Moreover, the surface morphology of trophoblastic epithelium was characterized by SEM imaging (Figure 3E). These data indicated the microscopic fibrous structure of microvilli covered on the placental barrier.

To assess the integrity of the formed barrier model, we compared the transfer of FITC‐dextran (*M*_w_ = 70 kDa) through the multilayered placental barrier, the trophoblastic epithelium and the porous membrane without cells (Figure 3F). The percentage of dye transfer through the membrane after 3 h was 33.3% for uncovered membrane, 3.3% for trophoblast layers and 0.3% for trophoblast cells cocultured with HUVECs. The results showed that the barrier permeability in co‐cultures is significantly lower than that in the single‐culture trophoblast layer, indicating the important role of co‐cultures in maintaining the integrity of the placental barrier.

### 3.3 Influence of fluid flow on the functionality of placental barrier

Human placental villi in vivo are exposed to low shear stress from maternal blood flow in the first trimester. Previous reports have demonstrated that flow shear stress (FSS) could induce microvilli formation in BeWo cells and enhance fusion of CT into ST in rabbit trophoblast stem cells.^32^ In order to investigate the effects of FSS on differentiation of hTSCs and function of placental barrier, we made comparison of the placental models under static and dynamic culture condition.

**FIGURE 4:**
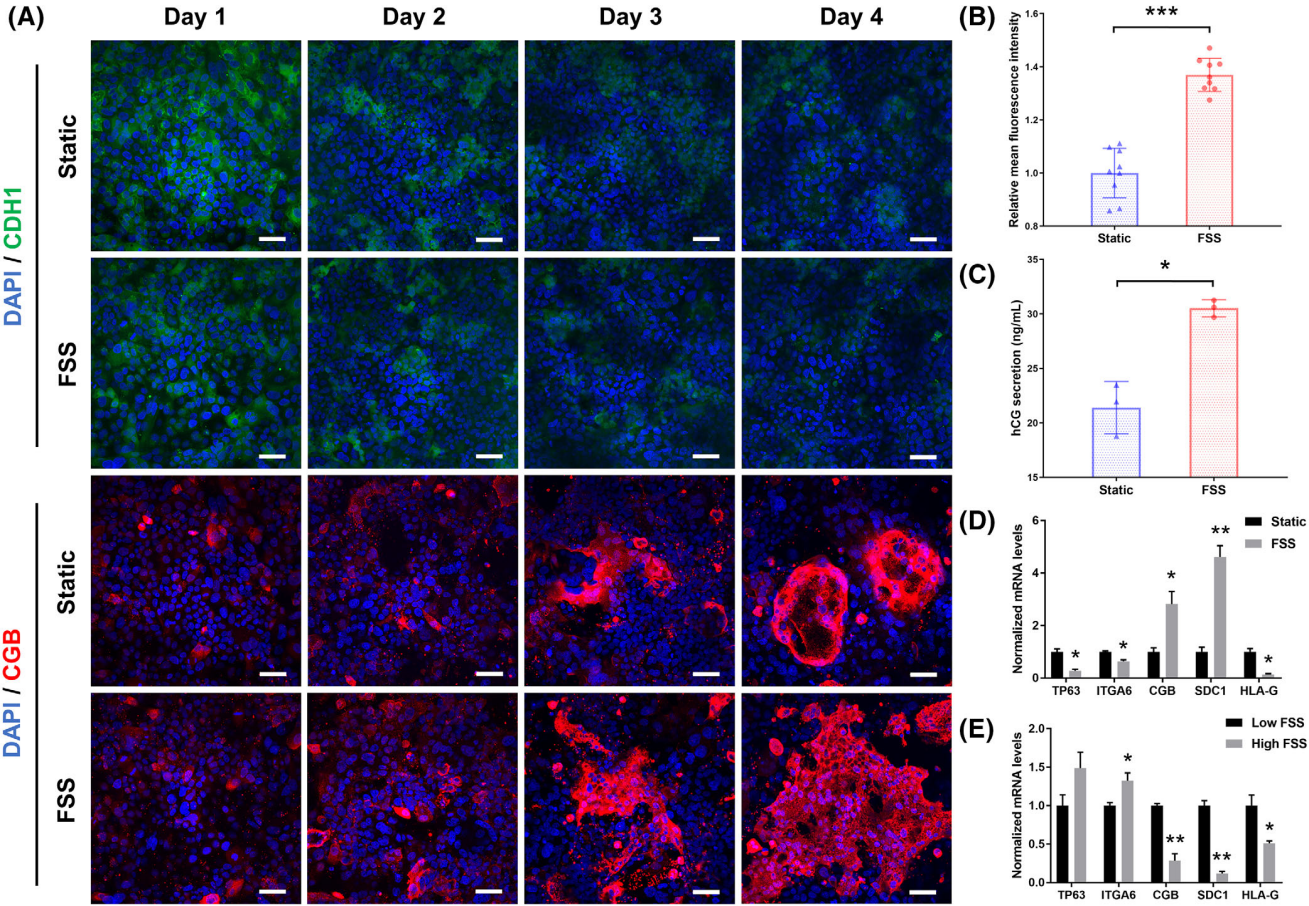
Effects of fluid flow on the trophoblast syncytialization. (A) Representative fluorescence image of the trophoblast layer stained with CDH1 antibody (green), CGB antibody (red) and DAPI (blue) under static or dynamic culture. Samples were collected every 24 h from day 1 to day 4. Scale bars are 50 μm. (B) Relative mean fluorescence intensity of CGB expression on the trophoblast layer under static or dynamic culture. The image‐based fluorescence intensity statistics were sampled from three different chips and each for three different areas. The areas were picked randomly. Image J software was used for statistical analysis. The data are presented as the mean ± SD. Data significance was assessed by unpaired two‐tailed Student's *t*‐test; ***p < 0.001. (C) hCG secretion by trophoblast cells collected from the placental barrier under static or dynamic culture. The concentration of hCG in culture medium was analysed by ELISA kit. The data are presented as the mean ± SD. Data significance was assessed by unpaired two‐tailed Student's *t*‐test; *p < 0.05. (D) Relative mRNA expression of TP63, ITGA6, CGB, SDC1 and HLA‐G in trophoblast cells collected under static or dynamic culture. mRNA expression normalized to GAPDH RNA level was analysed by real‐time PCR. The data are presented as the mean ± SEM from three independent experiments. Data significance was assessed by unpaired two‐tailed Student's *t*‐test; *p < 0.05, **p < 0.002.

We first examined the expression of CDH1 and CGB on trophoblast layers under static or dynamic culture from day 1 to day 4 by immunofluorescence. The representative images showed the decreased expression of CDH1 while enhanced expression of CGB over time under both static and perfused condition (Figure 4A). However, FSS‐exposed trophoblast cells showed significantly higher expression of CGB in contrast with the static group as shown in the immunostaining and mean fluorescence intensity analysis (Figure 4B). The secretion of hCG also showed a higher level in trophoblasts under perfusion culture (Figure 4C), indicating that FSS promoted the placental hormone synthesis and secretion. Then, we investigated the relative mRNA expression of markers related to trophoblast differentiation by RT‐qPCR. The data displayed upregulation of ST markers (CGB and SDC1), downregulation of CT markers (TP63 and ITGA6) and the EVT marker (HLA‐G) (Figure 4D), which confirmed the promotion effects of FSS on trophoblast syncytialization. Moreover, we explored the response of trophoblasts exposed to FSS at different levels. The result of RT‐qPCR showed that high FSS (100 μL/h, ~0.05 dyn/cm^2^) attenuated the differentiation of trophoblasts compared to the low FSS (10 μL/h, ~0.005 dyn/cm^2^), suggesting that a moderate shear stress was preferred for proliferation and differentiation of hTSCs in early pregnancy (Supplementary Figure 4). Taken together, these results demonstrated that physiologically relevant FSS could facilitate the differentiation of hTSCs into ST and hCG secretion of placental barrier during early gestation.

**FIGURE 5:**
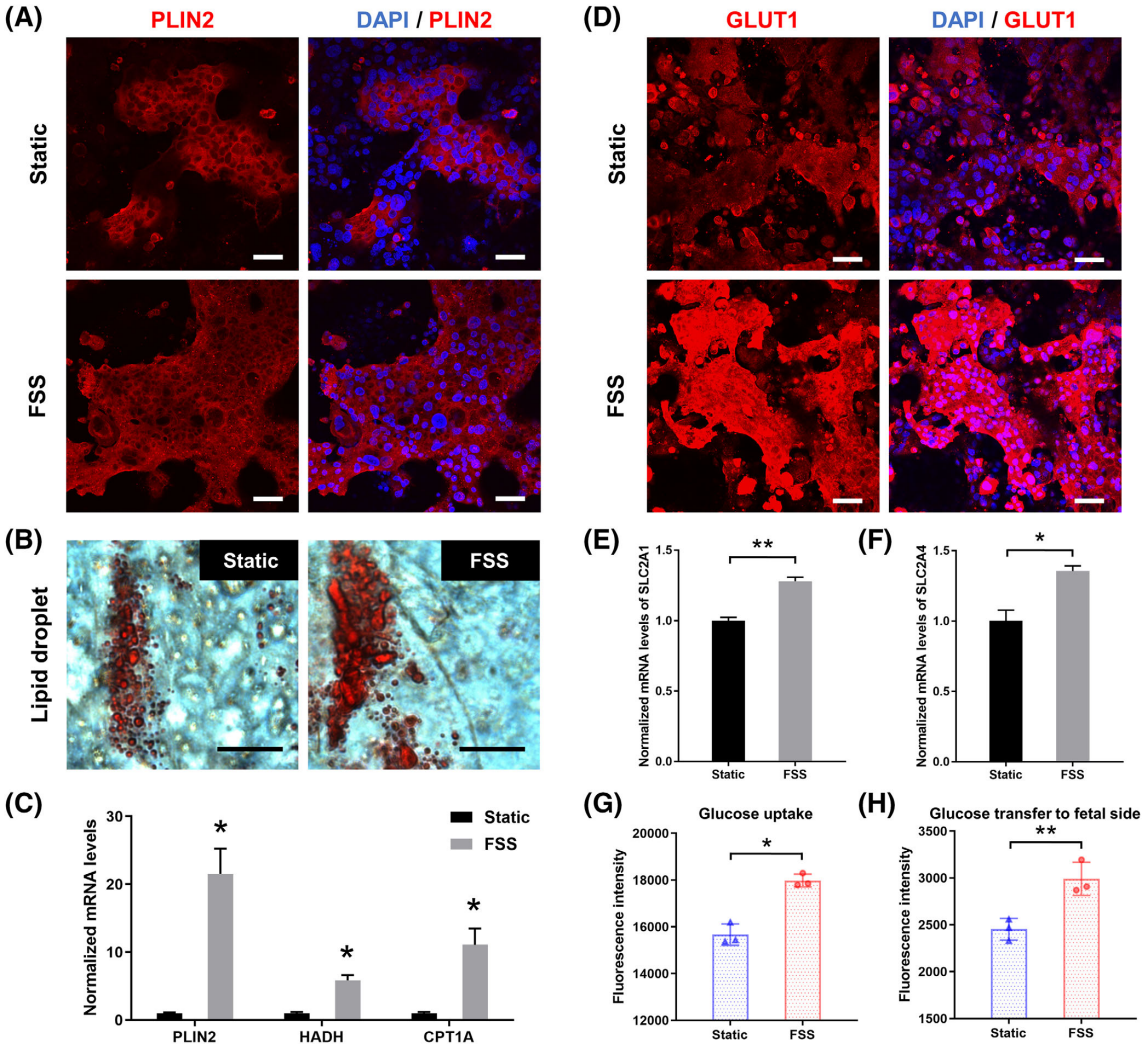
Characterization of lipid metabolism and glucose transport activity in trophoblasts under fluid flow conditions. (A) Representative fluorescence image of the trophoblast layer stained with PLIN2 antibody (red) and DAPI (blue) under static or dynamic culture. Scale bars are 50 μm. (B) Production of lipid droplets in trophoblastic epithelium under static or dynamic culture. The lipid droplets were stained by Oil Red O. Scale bars are 20 μm. (C) Relative mRNA expression of PLIN2, HADH and CPT1A in trophoblast cells collected under static or dynamic culture. mRNA expression normalized to GAPDH RNA level was analysed by real‐time PCR. The data are presented as the mean ± SEM from three independent experiments. Data significance was assessed by unpaired two‐tailed Student's *t*‐test; *p < 0.05. (D) Representative fluorescence image of the trophoblast layer stained with GLUT1 antibody (red) and DAPI (blue) under static or dynamic culture. Scale bars are 50 μm. (E, F) Relative mRNA expression of SLC2A1 and SLC2A4 in trophoblast cells collected under static or dynamic culture. mRNA expression normalized to GAPDH RNA level was analysed by real‐time PCR. The data are presented as the mean ± SEM from three independent experiments. Data significance was assessed by unpaired two‐tailed Student's *t*‐test; *p < 0.05, **p < 0.002. (G, H) Glucose uptake and transfer to fetal side of trophoblastic barrier under static or dynamic culture. The glucose transport activity was tested by 2‐NBDG uptake assays. The data are presented as the mean ± SD. Data significance was assessed by unpaired two‐tailed Student's *t*‐test; *p < 0.05, **p < 0.002.

We further assessed the changes of trophoblastic epithelium in lipid metabolism and glucose transport under perfusion culture. The lipid droplet coating protein perilipin 2 (PLIN2) plays an important role in lipid droplet accumulation.^33^ In contrast to the static group, FSS‐exposed trophoblast layer showed a larger area of PLIN2 expression with the similar cell density (Figure 5A). Moreover, the result of Oil red O staining showed a bigger size of lipid droplet formation in trophoblasts under fluid flow condition (Figure 5B), indicating that FSS enhanced the lipid droplet accumulation in trophoblast cells. Apart from PLIN2, the HADH and CPT1A genes, which are closely related to fatty acid catabolism, also showed higher expression in the FSS group as shown by RT‐qPCR (Figure 5C). As to the glucose transport, the immunofluorescence images showed significantly stronger expression of GLUT1 in FSS‐exposed trophoblastic epithelium compared to the static group (Figure 5D). Besides, trophoblasts under perfusion culture showed an increase in mRNA expression levels of GLUT1 (SLC2A1) and GLUT4 genes (SLC2A4) (Figure 5E,F), suggesting that FSS may facilitate the glucose transport through placental barrier. The 2‐NBDG based assay further confirmed that FSS‐exposed trophoblasts not only showed higher glucose uptake, but also allowed more glucose transfer to the fetal side (Figure 5G,H). Collectively, these results demonstrated the promotion effects of FSS on development of placental trophoblasts in lipid metabolism and glucose transport activity.

**FIGURE 6:**
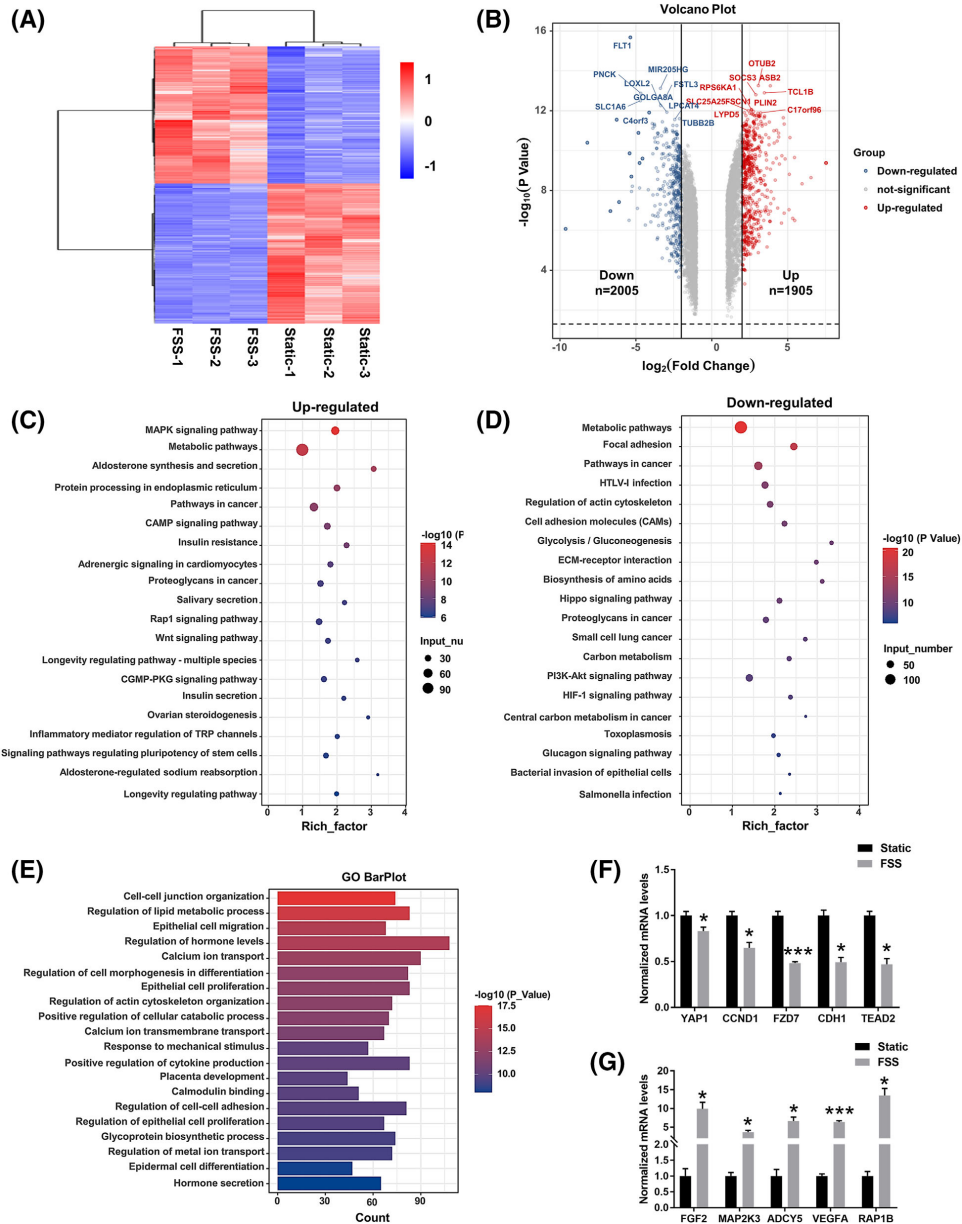
Profiling the effects of flow shear stress on trophoblastic epithelium by transcriptome analysis. (A) Hierarchical clustering heat map for DEGs between trophoblast cells under static or dynamic cultures. The gradient colour scale at the right top indicates the log2 (fold change) in the expression of the treatment case compared with the control case. (B) Volcano plots of the significantly DEGs between trophoblast cells under static or dynamic cultures. Genes differentially expressed with fold change over 2.0 and *p* < 0.05 were marked in colour. (C, D) KEGG functional classification of the DEGs between trophoblast cells under static or dynamic cultures. The colour of the dots represents the rich factor, while the size represents the input number of genes for each KEGG term. (E) GO analysis between trophoblast cells under static or dynamic cultures. (F, G) Validation of selected DEGs identified by RNA‐seq using RT‐qPCR. The expression values were normalized to GAPDH. Data were normalized against mono‐culture expression values and are shown as mean ± SEM; *p < 0.05, ***p < 0.001.

In order to further profile the effects of FSS on trophoblasts, we performed RNA sequencing analysis of trophoblasts from the placental barrier model at day 4. Hierarchical clustering of differentially expressed genes (DEGs) in the heatmap showed significant differences between trophoblasts under static and dynamic cultures (Figure 6A). Volcano plots showed that 2005 down‐regulated genes and 1965 up‐regulated genes among DEGs were notably modulated in FSS‐exposed trophoblast cells (Figure 6B). The top 10 down‐regulated and up‐regulated genes include SLC25A25, PNCK, and C4orf3 that are closely associated with the regulation of calcium signalling.^34^, ^35^, ^36^ Kyoto Encyclopedia of Genes and Genomes (KEGG) analysis was performed to identify enriched signalling pathways among significantly regulated DEGs (Figure 6C, D). The results showed that Hippo, MAPK and Rap1 signalling pathways were enriched in trophoblasts under fluid flow conditions. These pathways were related to the fusion of trophoblasts and calcium ion fluxes.^37^, ^38^, ^39^ Moreover, gene ontology (GO) analysis identified enhanced biological processes including calcium ion transport, calmodulin binding, epidermal cell differentiation and hormone secretion in FSS‐exposed trophoblasts (Figure 6E). The data above suggested that syncytialization of trophoblasts were probably facilitated by FSS through calcium‐related downstream pathways. RNA sequencing about genes related to Hippo signalling pathway (Figure 6F) and Rap1 signalling pathway (Figure 6G) were finally verified by RT‐qPCR.

### 3.4 Responses of placental barrier model to mono‐2‐ethylhexyl phthalate (MEHP) exposure

**FIGURE 7:**
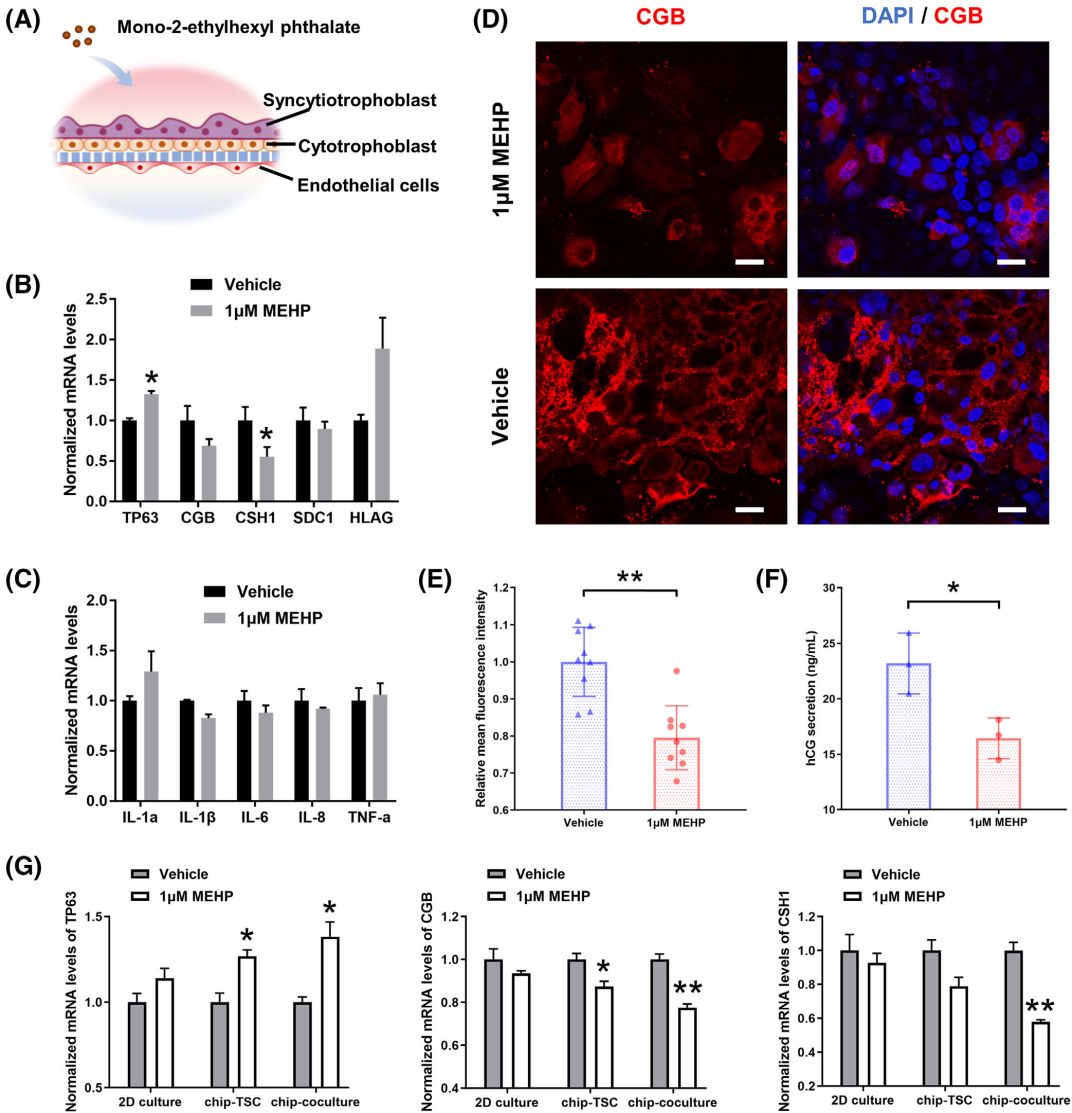
Responses of the placental barrier model to MEHP exposure. (A) Schematic of the placental barrier exposed to MEHP. MEHP were added into the upper channel to mimic the direct exposure in maternal blood. (B) Relative mRNA expression in trophoblast cells collected from the placental barrier model treated with vehicle or 1 μM MEHP. The data are presented as the mean ± SEM. Data significance was assessed by unpaired two‐tailed Student's *t*‐test; *p < 0.05. (C) Relative mRNA expression in human umbilical endothelial cells collected from the placental barrier model treated with vehicle or 1 μM MEHP. The data are presented as the mean ± SEM. Data significance was assessed by unpaired two‐tailed Student's *t*‐test. (D) Representative fluorescence image of the trophoblast layer stained with CGB antibody (red) and DAPI (blue) under treatment with 1 μM MEHP or vehicle. Scale bars are 20 μm. (E) Relative mean fluorescence intensity of CGB expression on the trophoblast layer treated with vehicle or 1 μM MEHP. The image‐based fluorescence intensity statistics were sampled from three different chips and each for three different areas. The areas were picked randomly. Image J software was used for statistical analysis. The data are presented as the mean ± SD. Data significance was assessed by unpaired two‐tailed Student's *t*‐test; **p < 0.002. (F) hCG secretion by trophoblast cells collected from the placental barrier model with vehicle or 1 μM MEHP treatment. The concentration of hCG in culture medium was analysed by ELISA kit. The data are presented as the mean ± SD. Data significance was assessed by unpaired two‐tailed Student's *t*‐test; *p < 0.05. (G) Relative mRNA expression in trophoblast cells collected from the 2D trophoblast cell model, trophoblastic epithelium‐on‐chip model (chip‐TSC) and placental barrier‐on‐chip model (chip‐coculture) treated with vehicle or 1 μM MEHP. The data are presented as the mean ± SEM. Data significance was assessed by unpaired two‐tailed Student's *t*‐test; *p < 0.05, **p < 0.002.

The placental development is susceptible to environmental toxicants, especially in early gestation.^40^ MEHP is one of the endocrine disrupting chemicals found in maternal blood, which is the primary metabolite of the common plasticizing agent di(2‐ethylhexyl) phthalate (DEHP). The concentration of MEHP in maternal and umbilical cord blood may reach to 1–40 μM.^41^ Several studies have reported that MEHP exposure was associated with disturbance of placental and fetal development.^42^, ^43^ To verify the irritability of this placental model in response to phthalates, we added MEHP in the trophoblast side on chip to mimic the environmental toxicants exposure in maternal circulatory system (Figure 7A).

We first examined the viability of hTSCs and HUVECs with MEHP exposure. The two types of cells were exposed to MEHP at different concentrations for 48 h in 96‐well plate, respectively. The result showed that the viability of hTSCs decreased with the dose increase of MEHP, while HUVECs were barely affected (Figure S4). Then, we tested the expression of genes related to hTSCs differentiation in trophoblasts exposed to MEHP at different dose levels. The data showed that 0.1, 1, and 10 μM MEHP inhibited the differentiation of hTSCs, but 100 μM MEHP promoted the expression of ST markers (Figure S5). Such a U‐shaped effect of MEHP was consistent with previous study.^41^ Based on the above results, we finally chose MEHP at 1 μM as the experimental condition on our placental model.

The human placental barrier models were treated with MEHP or vehicle (DMSO) for 48 h. Then, trophoblast cells and HUVECs were separately collected for RNA isolation and RT‐qPCR analysis. The data displayed downregulation of CGB and CSH1 genes while upregulation of TP63 and HLA‐G genes in trophoblasts, which demonstrated that low‐dose MEHP could suppress trophoblast syncytialization and hormone production (Figure 7B). The mRNA expression of inflammatory factors in HUVECs were also detected. Among them, only IL‐1a increased due to the exposure of MEHP, suggesting that 1 μM MEHP barely trigger inflammation on the fetal side (Figure 7C). To further verified the inhibitory effects of low‐dose MEHP in syncytialization process. We examined the expression of CGB on protein level by immunofluorescence. As shown in the fluorescence images (Figure 7D) and statistics of mean fluorescence intensity (Figure 7E), the expression of CGB decreased in MEHP‐exposed trophoblast layers. Moreover, the result of ELISA showed reduced hCG secretion in trophoblasts exposed to MEHP (Figure 7F). In order to verify the physiologically relevant responses of this placental model to MEHP exposure, we further compared the expression of genes related to trophoblast differentiation among 2D trophoblast cell model, trophoblast epithelium‐on‐chip model (chip‐TSC) and the placental barrier‐on‐chip model (chip‐coculture). As shown in Figure 7G, the chip‐coculture model showed more significant difference of genes expression compared with other groups, while the 2D cell model with MEHP exposure showed no significant difference of these genes expression. The data indicated that the placental chip model showed more sensitive and physiologically relevant responses to MEHP exposure than the 2D cell model. In conclusion, our placental model showed inhibited differentiation into ST and impaired production of pregnancy‐related hormones in response to the low‐dose MEHP exposure.

## 4 DISCUSSION

In this work, we constructed a novel placental barrier model derived from hTSCs by combining stem cell biology with bioengineering technology, which allows to recapitulate the key architecture and functions of human early placenta. The multilayered chip device enabled the coculture of trophoblasts and endothelial cells in a dynamic microenvironment. hTSCs differentiated into major trophoblast cell types including CT and ST, which could self‐assemble into bilayered trophoblastic epithelium with dense microvilli under fluid flow on chip. Moreover, the formed placental barrier exhibited increased expression of ST markers and enhanced barrier function under dynamic cultures, including hCG secretion, lipid metabolism and glucose transport activity. It recapitulates the key features of human early placental villi in vivo. RNA‐seq analysis revealed the enhanced trophoblast differentiation from hTSCs on chip, which was associated with shear stress, calcium and placental development signalling pathways. In addition, the established model exposed to low‐dose MEHP showed disturbed syncytialization process and hormone secretion of trophoblasts, which provides a proof‐of‐concept to assess the responses of human placenta to environmental exposure.

Placenta is an organ sensitive to mechanical forces, especially the fluid shear stress caused by maternal blood flow.^44^ Trophoblast cells are exposed to a broad range of shear stress value from 0.001 to 30 dyn/cm^2^ in different periods *in vivo*. In the first trimester, blood flow in uterine spiral arteries is blocked by extravillous trophoblastic plugs, which results in a small amount of maternal blood passed through the plugs into intervillous space.^45^ Therefore, trophoblasts subject to a relatively low FSS during early gestation. In this study, we built the placental barrier on chip using a shear stress of ~0.005 dyn/cm^2^ at a perfusion speed of 10 μL/h to mimic the low FSS. The results demonstrated that low FSS was beneficial to syncytialization and enhanced functions of placental barrier, including lipid metabolism and glucose transport activity. Previous study reported similar effects of FSS on rabbit trophoblast stem cells,^46^ but no works have been devoted to engineer placental barrier model using hTSCs. In addition, we confirmed that low FSS (~0.005 dyn/cm^2^) is more favourable for the differentiation of trophoblasts compared to high FSS (~0.05 dyn/cm^2^). The results suggested that our placental model recapitulated the key structural and functional features of human early placental development in a physiologically relevant microenvironment.

According to the transcriptome analysis, our placental barrier model showed a crucial and beneficial role of fluid flow in syncytialization of trophoblasts. The Hippo signalling pathway‐related genes YAP1, TEAD2 and CCND1 showed down‐regulated expressions, which are associated with trophoblasts stemness and proliferation.^47^, ^48^, ^49^ While the Rap1 signalling pathway‐related genes FGF2, MAP2K3 and ADCY7 showed up‐regulated expressions, which are related to the regulation of trophoblast syncytialization.^50^, ^51^, ^52^ These results revealed that fluid flow facilitated the differentiation of hTSCs into ST. Among the top 10 up‐regulated genes in volcano plots, SLC25A25, one of the family of calcium‐binding mitochondrial carriers, acts as the downstream of TRPP2 in cellular metabolism through mediating calcium signals.^36^ It indicated that the activity of calcium transporter in trophoblast cells may be related to TRPP2 upregulation. Similarly, TRPV6 was reported to regulate microvilli formation in trophoblast cells through calcium ion influx in response to FSS.^32^ These findings may provide new insights into the relation between fluid flow and trophoblast differentiation.

The extensive use of DEHP in polyvinyl chloride materials caused a wide distribution of phthalates in the environment. MEHP, a metabolism of DEHP in human bodies, has been reported to be more toxic than its precursor.^53^ Previous studies have identified the side effects of MEHP on trophoblast differentiation and syncytialization.^41^ In our model, we demonstrated a U‐shaped dose–response effect of MEHP on hTSCs differentiation, which is similar to previous study of primary trophoblasts. The results of comparison with 2D model suggested that intercellular interaction between endothelium and trophoblasts might make trophoblastic epithelium more sensitive to MEHP exposure, although MEHP barely showed direct toxicity to HUVECs. Moreover, our study indicated that low‐dose MEHP promoted the differentiation of trophoblast cells into EVT, which may be associated with the abnormal trophoblast migration. These responses of the placental model to endocrine disrupting chemicals are often related to placental dysfunctions and disrupted placental development at early stages. It also demonstrated that this bioengineered model could provide a new platform for the investigation of environmental toxicants exposure in human early placenta.

Despite the potential applications of our model, there is still space for improvement. In this work, PDMS is the main material for fabricating chip device, but it may adsorb organic molecules, such as hormones or chemicals, impeding precise drug testing and quantitative response. Although we modified the surface of PDMS channels with PF127 to reduce adsorption of molecules, the chip materials for more biocompatibility and less nonspecific adsorption is needed to be considered. In addition, given the complexity of human placenta, the incorporation of more physiologically relevant placental cells could further improve the functions of placental model. For example, the interactions between trophoblasts and immune cells including natural killer cell and Hofbauer cell may contribute to construct more predictive models and reflect more accurate responses to external stimuli at maternal‐fetal interface. We envision that other bioengineered approaches or microfluidic elements could be incorporated to advance the development of placental models with high fidelity, thereby contributing to their applications in studies of human reproductive health and disease.

## AUTHOR CONTRIBUTIONS

Jianhua Qin conceived the study and revised the manuscript. Rongkai Cao performed most of the experiments and writed the manuscript.Yaqing Wang designed the experiments and writed the manuscript. Jiayue Liu analysed the data of RNA sequencing. Lujuan Rong provided the human trophoblast stem cells.

## FUNDING INFORMATION

This research was supported by the Strategic Priority Research Program of the Chinese Academy of Sciences (No. XDA16020900, XDA16021300), National Key R&D Program of China (No. 2022YFA1104700), the National Natural Science Foundation of China (No. 31971373, 32171406), Yunnan Key Research and Development Program (202003AD150009), Project from China National Tobacco Corporation (110202102015), and Scientific Research Program of Innovation Platform in State Tobacco Monopoly Administration (312021AW0).

## CONFLICT OF INTEREST STATEMENT

The authors declare that they have no conflict of interest.

## Supporting information


**Figure S1.** Global view of the hTSCs derived placental barrier‐on‐a‐chip model. (A) Fluorescence image of the trophoblastic epithelium stained with CDH1 antibody (green), CGB antibody (red) and DAPI (blue). Scale bars are 200 μm. (B) Fluorescence image of the whole placental barrier stained with CK7 antibody (green) and DAPI (blue). HUVECs were labelled with RFP (red). Scale bars are 200 μm.
**Figure S2.** Z‐axis scanning of the trophoblastic epithelium on day 0 and day 4. (A) Representative three sides image of the trophoblastic epithelium on day 0 stained with phalloidin (F‐actin, green) and DAPI (blue). Scale bars are 20 μm. (B) Representative three sides image of the trophoblastic epithelium on day 4 stained with phalloidin (F‐actin, green) and DAPI (blue). Scale bars are 20 μm.
**Figure S3.** Relative mRNA expression of TP63, ITGA6, CGB, SDC1 and HLA‐G in trophoblast cells cultured under low (10 μL/h) or high (100 μL/h) flow shear stress. mRNA expression normalized to GAPDH RNA level was analysed by real‐time PCR. The data are presented as the mean ± SEM from three independent experiments. Data significance was assessed by unpaired two‐tailed Student's *t*‐test; *p < 0.05, **p < 0.002.
**Figure S4.** Viability analysis of hTSCs and HUVECs exposed to MEHP at different concentration. (A) Viability of hTSCs treated with 0 (vehicle, DMSO), 0.1, 1, 10, 50 and 100 μM MEHP for 48 h. The data are presented as the mean ± SD. (B) Viability of HUVECs treated with 0, 0.1, 1, 10, 50 and 100 μM MEHP for 48 h. The data are presented as the mean ± SD.
**Figure S5.** RNA expression of ST markers in trophoblast cells exposed to MEHP at different concentration. (A) Relative mRNA expression of CGB in trophoblast cells treated with 0 (vehicle, DMSO), 0.1, 1, 10 and 100 μM MEHP for 48 h. The data are presented as the mean ± SEM. (B) Relative mRNA expression of SDC1 in trophoblast cells treated with 0, 0.1, 1, 10 and 100 μM MEHP for 48 h. The data are presented as the mean ± SEM.
**Table S1.** Primers pairs used to detect the mRNA expression.

## Data Availability

All relevant data are available in the manuscript or Supporting Information. All the RNA‐seq raw data have been deposited on SRA under the accession number PRJNA916373.
