# Supplementary material for: Self‐assembled human placental model from trophoblast stem cells in a dynamic organ‐on‐a‐chip system
Source: Cell Prolif. 2023 May 17;56(5):e13469. doi: 10.1111/cpr.13469 (PMC10212715; doi:10.1111/cpr.13469)
Supplement: Supplementary file 1 — Figure S1. Global view of the hTSCs derived placental barrier‐on‐a‐chip model. (A) Fluorescence image of the trophoblastic epithelium stained with CDH1 antibody (green), CGB antibody (red) and DAPI (blue). Scale bars are 200 μm. (B) Fluorescence image of the whole placental barrier stained with CK7 antibody (green) and DAPI (blue). HUVECs were labelled with RFP (red). Scale bars are 200 μm. Figure S2. Z‐axis scanning of the trophoblastic epithelium on day 0 and day 4. (A) Representative three sides image of the trophoblastic epithelium on day 0 stained with phalloidin (F‐actin, green) and DAPI (blue). Scale bars are 20 μm. (B) Representative three sides image of the trophoblastic epithelium on day 4 stained with phalloidin (F‐actin, green) and DAPI (blue). Scale bars are 20 μm. Figure S3. Relative mRNA expression of TP63, ITGA6, CGB, SDC1 and HLA‐G in trophoblast cells cultured under low (10 μL/h) or high (100 μL/h) flow shear stress. mRNA expression normalized to GAPDH RNA level was analysed by real‐time PCR. The data are presented as the mean ± SEM from three independent experiments. Data significance was assessed by unpaired two‐tailed Student's t‐test; *p < 0.05, **p < 0.002. Figure S4. Viability analysis of hTSCs and HUVECs exposed to MEHP at different concentration. (A) Viability of hTSCs treated with 0 (vehicle, DMSO), 0.1, 1, 10, 50 and 100 μM MEHP for 48 h. The data are presented as the mean ± SD. (B) Viability of HUVECs treated with 0, 0.1, 1, 10, 50 and 100 μM MEHP for 48 h. The data are presented as the mean ± SD. Figure S5. RNA expression of ST markers in trophoblast cells exposed to MEHP at different concentration. (A) Relative mRNA expression of CGB in trophoblast cells treated with 0 (vehicle, DMSO), 0.1, 1, 10 and 100 μM MEHP for 48 h. The data are presented as the mean ± SEM. (B) Relative mRNA expression of SDC1 in trophoblast cells treated with 0, 0.1, 1, 10 and 100 μM MEHP for 48 h. The data are presented as the mean ± SEM. Table S1. Primers pair [file CPR-56-e13469-s001.docx]

**Supporting information**


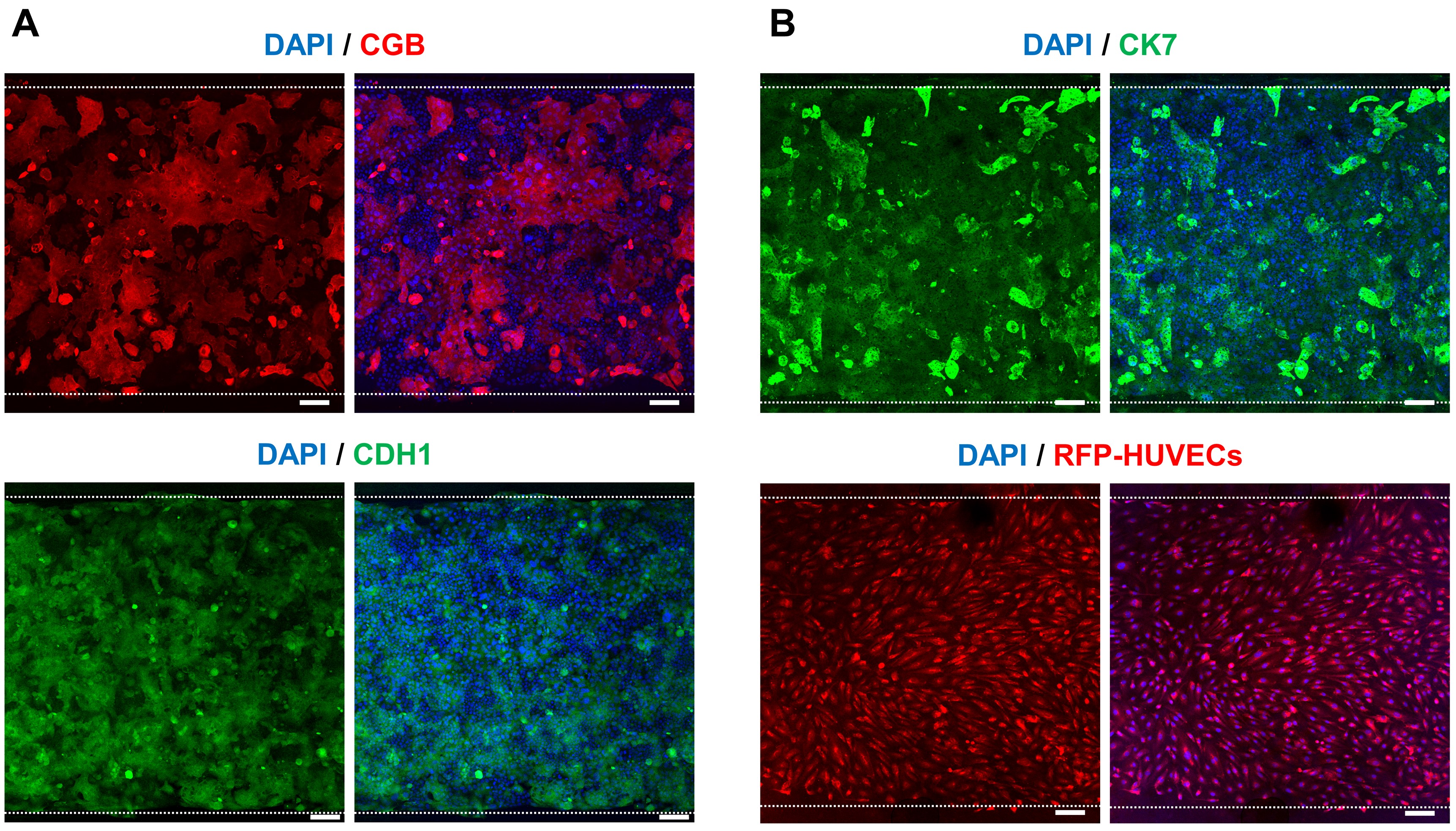
**Fig S1.** Global view of the hTSCs derived placental barrier-on-a-chip model. (A) Fluorescence image of the trophoblastic epithelium stained with CDH1 antibody (green), CGB antibody (red) and DAPI (blue). Scale bars are 200μm. (B) Fluorescence image of the whole placental barrier stained with CK7 antibody (green) and DAPI (blue). HUVECs were labeled with RFP (red). Scale bars are 200μm.


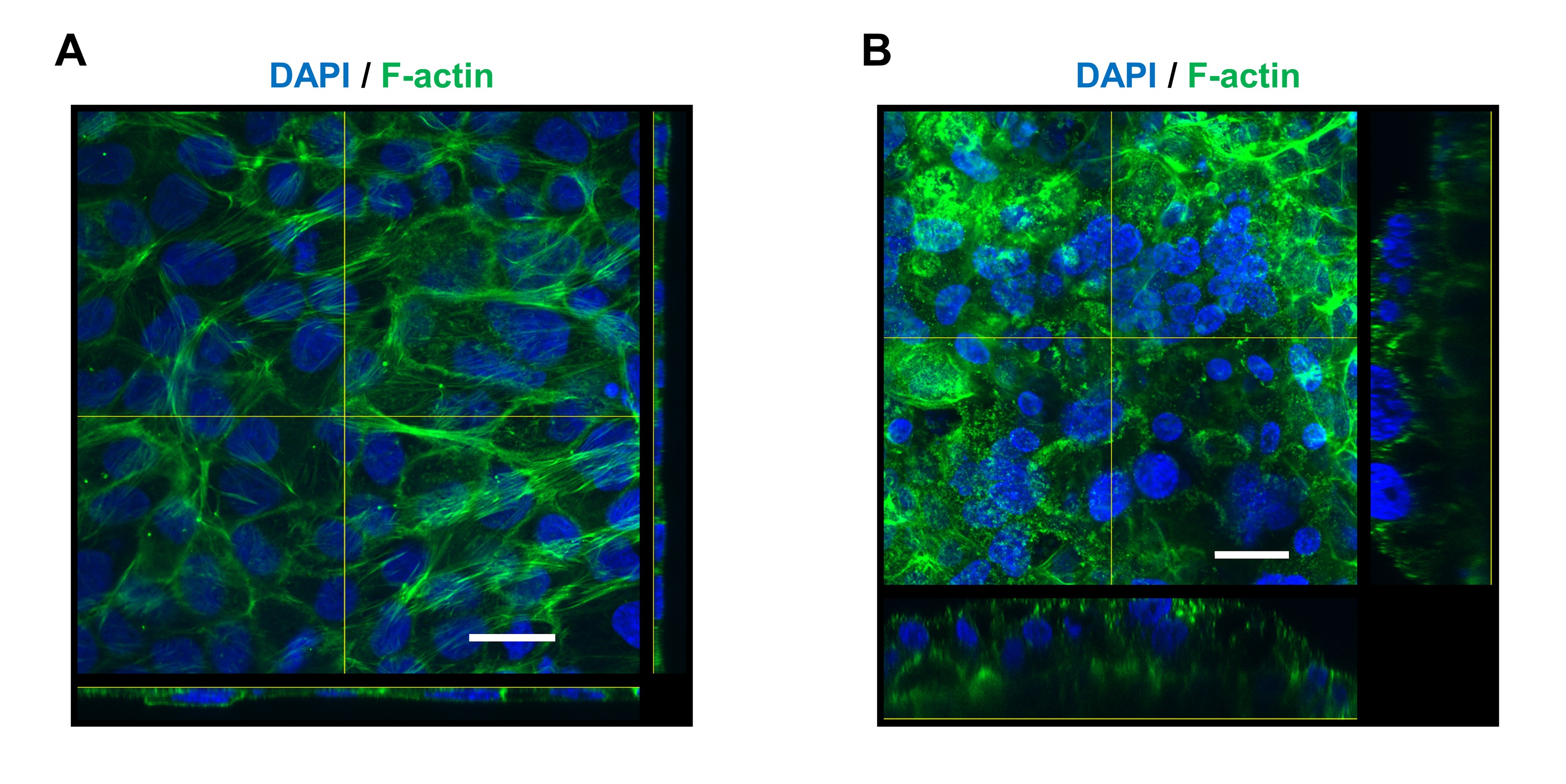
**Fig S2.** Z-axis scanning of the trophoblastic epithelium on day 0 and day 4. (A) Representative threesides image of the trophoblastic epithelium on day 0 stained with phalloidin (F-actin, green) and DAPI (blue). Scale bars are 20μm. (B) Representative threesides image of the trophoblastic epithelium on day 4 stained with phalloidin (F-actin, green) and DAPI (blue). Scale bars are 20μm.

**Fig S3.** Relative mRNA expression of TP63, ITGA6, CGB, SDC1 and HLA-G in trophoblast cells cultured under low (10μL/h) or high (100μL/h) flow shear stress. mRNA expression normalized to GAPDH RNA level was analyzed by real-time PCR. The data are presented as the mean±SEM from three independent experiments. Data signiﬁcance was assessed by unpaired two-tailed Student’s t-test; *P < 0.05, **P < 0.002.


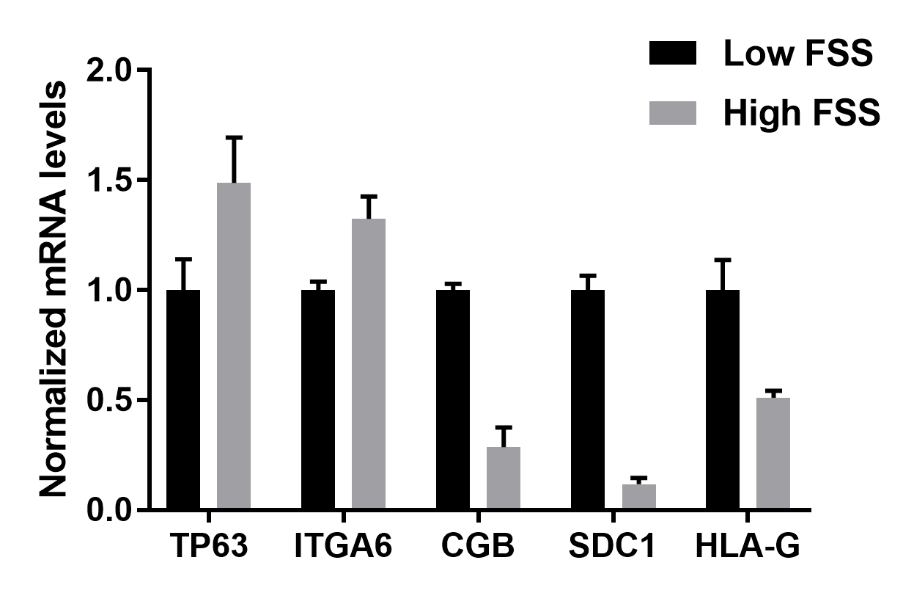


*

**

**

*


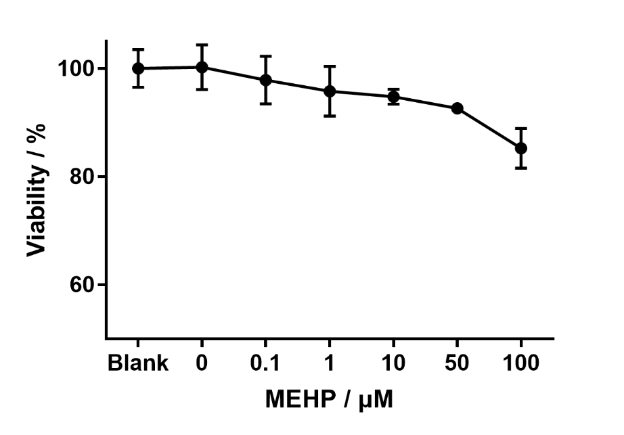

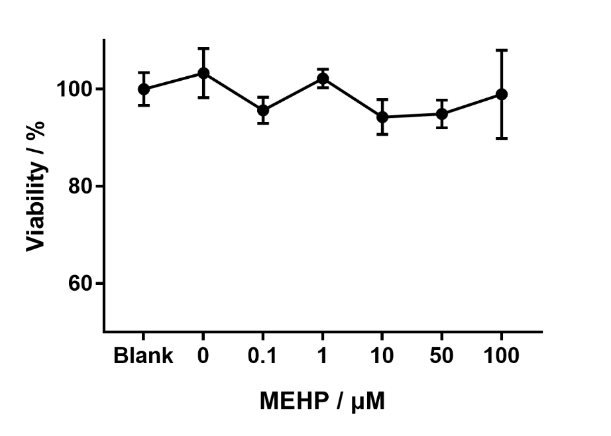


**A**

**B**

**Fig S4.** Viability analysis of hTSCs and HUVECs exposed to MEHP at different concentration. (A) Viability of hTSCs treated with 0 (vehicle, DMSO), 0.1, 1, 10, 50 and 100μM MEHP for 48h. The data are presented as the mean±SD. (B) Viability of HUVECs treated with 0, 0.1, 1, 10, 50 and 100μM MEHP for 48h. The data are presented as the mean±SD.

**A**

**B**


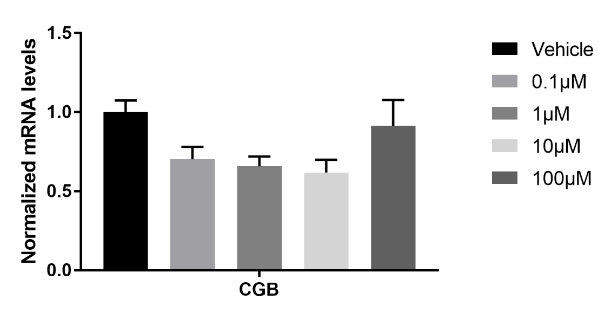

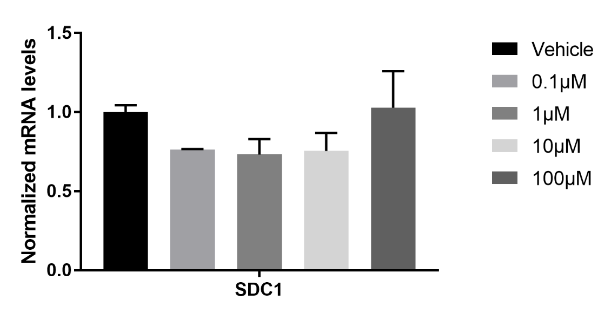


**Fig S5.** RNA expression of ST markers in trophoblast cells exposed to MEHP at different concentration. (A) Relative mRNA expression of CGB in trophoblast cells treated with 0 (vehicle, DMSO), 0.1, 1, 10 and 100μM MEHP for 48h. The data are presented as the mean±SEM. (B) Relative mRNA expression of SDC1 in trophoblast cells treated with 0, 0.1, 1, 10 and 100μM MEHP for 48h. The data are presented as the mean±SEM.

**Table S1**. Primers pairs used to detect the mRNA expression

| **Primer name** | **Sequence** |
| --- | --- |
| GAPDH | Fw 5′-CAATGACCCCTTCATTGACC-3′  Rv 5′-GACAAGCTTCCCGTTCTCAG-3′ |
| TP63 | Fw 5′-GGACCAGCAGATTCAGAACGG -3′  Rv 5′-AGGACACGTCGAAACTGTGC-3′ |
| ITGA6 | Fw 5′-GAGCTTTTGTGATGGGCGATT-3′  Rv 5′-CTCTCCACCAACTTCATAAGGC-3′ |
| CGB | Fw 5′-ACCCTGGCTGTGGAGAAGGAG-3′  Rv 5′-ATGGACTCGAAGCGCACATCG-3′ |
| SDC1 | Fw 5′-CTTCACACTCCCCACACAGA-3′  Rv 5′-GTATTCTCCCCCGAGGTTTC-3′ |
| CSH1 | Fw 5′-CATGACTCCCAGACCTCCTTCT-3′  Rv 5′-ATTTCTGTTGCGTTTCCTCCAT-3′ |
| HLA-G | Fw 5′-GAGGAGACACGGAACACCAAG-3′  Rv 5′-GTCGCAGCCAATCATCCACT-3′ |
| PLIN2 | Fw 5′-GCTGAGCACATTGAGTCACG-3′  Rv 5′-TGGTACACCTTGGATGTTGG-3′ |
| HADH | Fw 5′-TGTCGGACTGGATACTACGA-3′  Rv 5′-GATGGGCTGGGCTGATGTAA-3′ |
| CPT1A | Fw 5′-CCTACCACGGGTGGATGTTC-3′  Rv 5′-CAACATGGGTTTTCGGCCTG-3′ |
| SLC2A1 | Fw 5′-ATTGGCTCCGGTATCGTCAAC-3′  Rv 5′-GCTCAGATAGGACATCCAGGGTA-3′ |
| SLC2A4 | Fw 5′-GCCATGAGCTACGTCTCCATT-3′  Rv 5′-GGCCACGATGAACCAAGGAA-3′ |
| YAP1 | Fw 5′-TAGCCCTGCGTAGCCAGTTA-3′  Rv 5′-TCATGCTTAGTCCACTGTCTGT-3′ |
| CCND1 | Fw 5′-GCTGCGAAGTGGAAACCATC-3′  Rv 5′-CCTCCTTCTGCACACATTTGAA-3′ |
| FZD7 | Fw 5′-GTGCCAACGGCCTGATGTA-3′  Rv 5′-AGGTGAGAACGGTAAAGAGCG-3′ |
| CDH1 | Fw 5′-CGAGAGCTACACGTTCACGG-3′  Rv 5′-GGGTGTCGAGGGAAAAATAGG-3′ |
| TEAD2 | Fw 5′-CTTCGTGGAACCGCCAGAT-3′  Rv 5′-GGAGGCCACCCTTTTTCTCA-3′ |
| FGF2 | Fw 5′-AGTGTGTGCTAACCGTTACCT-3′  Rv 5′-ACTGCCCAGTTCGTTTCAGTG-3′ |
| MAP2K3 | Fw 5′-GAGGGAGACGTGTGGATCTG-3′  Rv 5′-CCGCACGATAGACACAGCAAT-3′ |
| ADCY5 | Fw 5′-TCTCCTGCACCAACATCGTG-3′  Rv 5′-CATGGCAACATGACGGGGA-3′ |
| VEGFA | Fw 5′-AGGGCAGAATCATCACGAAGT-3′  Rv 5′-AGGGTCTCGATTGGATGGCA-3′ |
| RAP1B | Fw 5′-AGCAAGACAATGGAACAACTGT-3′  Rv 5′-TGCCGCACTAGGTCATAAAAG-3′ |
| IL-1α | Fw 5′-CGCCAATGACTCAGAGGAAGA-3′  Rv 5′-AGGGCGTCATTCAGGATGAA-3′ |
| IL-1β | Fw 5′-AATCTGTACCTGTCCTGCGTGTT-3′  Rv 5′-TGGGTAATTTTTGGGATCTACACTCT-3′ |
| IL-6 | Fw 5′-GGTACATCCTCGACGGCATCT-3′  Rv 5′-GTGCCTCTTTGCTGCTTTCAC-3′ |
| IL-8 | Fw 5′-CTTGGCAGCCTTCCTGATTT-3′  Rv 5′-TTCTTTAGCACTCCTTGGCAAAA-3′ |
| TNF-α | Fw 5′-CCCAGGGACCTCTCTCTAATC-3′  Rv 5′-ATGGGCTACAGGCTTGTCACT-3′ |
